# Electrical Brain Responses to an Auditory Illusion and the Impact of Musical Expertise

**DOI:** 10.1371/journal.pone.0129486

**Published:** 2015-06-12

**Authors:** Christos I. Ioannou, Ernesto Pereda, Job P. Lindsen, Joydeep Bhattacharya

**Affiliations:** 1 Department of Psychology, Goldsmiths, University of London, London, United Kingdom; 2 Institute of Music Physiology and Musicians' Medicine, Hannover University of Music, Drama and Media, Hanover, Germany; 3 Electrical Engineering and Bioengineering Group, Department of Industrial Engineering, University of La Laguna, Tenerife, Spain; 4 Institute of Biomedical Technology (CIBICAN), University of La Laguna, Tenerife, Spain; ARC Centre of Excellence in Cognition and its Disorders (CCD), AUSTRALIA

## Abstract

The presentation of two sinusoidal tones, one to each ear, with a slight frequency mismatch yields an auditory illusion of a beating frequency equal to the frequency difference between the two tones; this is known as binaural beat (BB). The effect of brief BB stimulation on scalp EEG is not conclusively demonstrated. Further, no studies have examined the impact of musical training associated with BB stimulation, yet musicians' brains are often associated with enhanced auditory processing. In this study, we analysed EEG brain responses from two groups, musicians and non-musicians, when stimulated by short presentation (1 min) of binaural beats with beat frequency varying from 1 Hz to 48 Hz. We focused our analysis on alpha and gamma band EEG signals, and they were analysed in terms of spectral power, and functional connectivity as measured by two phase synchrony based measures, phase locking value and phase lag index. Finally, these measures were used to characterize the degree of centrality, segregation and integration of the functional brain network. We found that beat frequencies belonging to alpha band produced the most significant steady-state responses across groups. Further, processing of low frequency (delta, theta, alpha) binaural beats had significant impact on cortical network patterns in the alpha band oscillations. Altogether these results provide a neurophysiological account of cortical responses to BB stimulation at varying frequencies, and demonstrate a modulation of cortico-cortical connectivity in musicians' brains, and further suggest a kind of neuronal entrainment of a linear and nonlinear relationship to the beating frequencies.

## Introduction

When two sinusoidal tones with a slight frequency mismatch (i.e. 200 and 210 Hz) are separately presented to each ear, the listener perceives a beating frequency equal to the frequency mismatch between the two tones (i.e. 10 Hz); this is termed binaural beating [1, 2]. The two tones combined wax and wane as the two frequencies come in and out of phase with one another, and this phase interference produces an amplitude-modulated standing wave, the binaural beat (BB). This beat is not a physical property of the presented sound but a subjectively perceived auditory illusion, which has its origin in the brainstem’s superior olivary nucleus [3, 4]. BBs are perceived for low frequency mismatch (< 100 Hz) and lower carrier frequencies (<1 kHz) [1]; phase locking in most mammalian brains becomes less precise at carrier frequencies above 1 kHz and disappears completely at frequencies above 5 kHz [5, 6].

BB stimulation is assumed to drive and evoke neuronal responses observable on scalp EEG as steady-state responses (SSRs) over a temporal window [7–10]. For example, BB at 40 Hz elicited a SSR over fronto-central brain regions [8]. Karino *et al*. [7] reported SSRs evoked by BBs of 4 Hz and 6.66 Hz that were localized in the temporal, parietal and frontal regions. The authors further suggested the possible cognitive encoding of BBs at a cortical level as the phases of the SSRs showed large variability. Pratt *et al*. [11] studied event-related-potentials (ERP) of two BBs at 3 Hz and 6 Hz, with two base frequencies, 250 Hz and 2000 Hz, and reported distinct event-related-potential (ERP) components such as P50, N100, and P200 following tone-onsets, with larger brain responses to the lower base frequency and to the lower BB frequency. Draganova *et al*. [10] showed a 40 Hz auditory steady-state response (ASSR) in the auditory cortices during 40 Hz BB stimulation. All these studies suggest that brain activity corresponding to this illusory auditory beats can be identified from, and systematically affect, EEG scalp recordings. However, we have noticed three big gaps as follows in the neuroscientific literature on BB.

First, most studies have looked only at a limited number of frequency mismatch. The large-scale neural oscillatory responses to BB frequencies varying systematically over a relative wide range have not been characterized. We believe this is essential as using a range of BBs would allow us to study the effect of cross-frequency coupling in large scale brain responses. The SSRs primarily represent the interaction between neuronal assemblies and external stimulation with identical frequency, but the cross-frequency coupling suggests a more generalized interaction between neuronal oscillations and stimulating frequency. Indeed cross-frequency coupling at the neuronal level has been demonstrated to provide a general mechanism of functional integration at multiple spatiotemporal scales [12].

Second, no study, to our knowledge, has investigated the cortical network patterns against BB stimulation, yet an increasing body of evidence suggests that functional co-operation among near and distant brain areas underlie almost any sensory and cognitive processing [13, 14]. Understanding information processing at the cortical network level provides novel insight into the possible propagation of the auditory stimulation and the induction of the illusory beat.

Finally, little is known about the individual differences in the brain responses to BB stimulation. A recent attempt in this direction was made by Goodin and colleagues [15] who investigated EEG spectral power in thirty three participants exposed to BB in either theta (7 Hz) or beta (16 Hz) frequency for a period of 2 minute and measured the personality traits by standard Big Five Factor model; no significant relationships were found between BB-related EEG responses and Big Five personality traits. Instead of looking for personality dependent differences, a more pragmatic and useful approach would be to investigate training related differences. In this context, musicians are an important group to study as the musician's brain is a robust model of neuroplasticity [16, 17], and long-term musical training leads to 'auditory fitness', i.e. an enhanced processing of auditory information [18].

The current study was designed to address these issues by recording electrical brain responses as measured by high-density EEG signals obtained from healthy human adults, both musicians and non-musicians, when stimulated by a range of BBs from low frequency delta band to high frequency gamma band. We analysed the EEG signals in terms of neuronal oscillatory activity as measured by spectral power, and phase synchronization as measured by mean phase coherence [19] and phase lag index [20]. Further, we also characterized the underlying network pattern by measures based on graph theory approach [21].

In this study, we strategically focused our analysis on the neuronal oscillations in the alpha and gamma frequency bands of EEG signals for the following reasons. Firstly, large scale brain oscillations in the alpha band (8–12 Hz) are the most studied and understood brain rhythm [22] and have been found ubiquitously in most cortical and subcortical areas [23]. Further, when humans are visually stimulated by flickering stimuli, steady state oscillations in the alpha band and its higher harmonics exhibited most resonant effect [24]. So it would be important to investigate whether alpha oscillations show similar resonance effect in auditory domain. This is in clear alignment with the growing interest on the importance of studying alpha rhythms in audition (see for a review, [25]). Furthermore, alpha oscillations are crucially associated with the perception of various types of illusions, i.e. examples of perceptual accounts that cannot be explained by physical properties of the stimuli, from visual (e.g., wagon wheel illusion [26]), auditory (e.g., tinnitus [27]), to multisensory (e.g., double flash illusion [28]). As alpha oscillations reflect the degree of excitability of sensory cortices, we expected that it would be associated with the perception of the binaural beat, an auditory illusion. Second, there has been increasing evidence that the degree of neuronal synchronization in the gamma band (30 Hz and above) is significantly larger in trained musicians as compared to non-musicians during music related task processing [29–31]. Further, enhanced neuronal synchronization in the gamma band has also been observed in other expert groups like long-term meditators [32], proficient bilinguals [33], professional artists [34]. Considering the widespread role of gamma band in providing a general framework for cortical computation [35] and its possible modulation with neuroplasticity, we expected that the gamma band synchrony would be differentially involved between the musicians and non-musicians during the perception of illusory binaural beat.

Therefore, we had two specific predictions: (i) across both groups, EEG alpha band power would be largest when stimulated by BBs belonging to alpha band, and (ii) as compared to non-musicians, musicians would produce a larger network response against binaural beat in the gamma band.

## Materials and Methods

### Participants

Sixteen musicians (6 males, age 25.5 ± 3.18 years) and sixteen non-musicians (7 males, age 26.1 ± 3.82 years) participated in the study. All but one non-musician were right-handed. No participants reported any auditory deficits or neurological disorders. Our musicians reported an average of 18.4 (± 4.3) years of active engagement with a musical instrument and 14 (± 3.40) years of formal training on a solo instrument. They practiced their principal instrument an average of 21.59 (± 11.42) hours per week with 81.3% reported being professional musicians. All participants were kept blind to the real purposes of the experiment. They gave their written informed consent before the start of the experiment. The experimental protocol was approved by the Ethics Committee of the Department of Psychology at Goldsmiths, and the experiment was conducted in accordance with the Declaration of Helsinki.

### Stimuli

Auditory stimuli were presented in 34 blocks of 2 min and 20 s each. Each block consisted of a silent period of 20 s followed by two auditory conditions of 1 min each: the *non-binaural beat* (NB) and the *binaural beat* (BB). For both auditory conditions (NB and BB), the base frequency (*f*_*b*_) was 200 Hz. This selection was made after recent studies on BB stimulation using *f*_*b*_ between 100–300 Hz [7, 11, 36]. For the NB condition, both ears received auditory pure tones of 200 Hz across all blocks. For the BB condition, the left ear received an auditory pure tone of 200 Hz and the right ear an auditory pure tone of 200+*f*_*bb*_ Hz (*f*_*bb*_ = beat frequency) with *f*_*bb*_ varying with a step size of 1 Hz in the range of 1 Hz to 20 Hz and with a step size of 2 Hz from 20 Hz to 48 Hz. Based on the values of the beat frequency (*f*_bb_) lying within the standard EEG frequency bands [37], we formed five groups of BB stimuli as follows: delta-BB (1 Hz ≤ *f*_bb_ ≤ 4 Hz), theta-BB (5 Hz ≤ *f*_bb_ ≤ 8 Hz), alpha-BB (9 Hz ≤ *f*_bb_ ≤ 12 Hz), beta-BB (13 Hz ≤ *f*_bb_ ≤ 30 Hz), and gamma-BB (32 Hz ≤ *f*_bb_ ≤ 48 Hz). Following this grouping, we had four blocks each for delta-BB, theta-BB and alpha-BB, eight blocks for beta-BB and finally fourteen blocks for gamma-BB. Auditory stimuli were presented through a Philips in-ear headphone with rubber caps that reduced external noise, ensured a precise fit, and decreased effective surface for bone conduction [38, 39]. The volume of the auditory stimuli was self-adjusted a priori by each participant and was kept at constant level throughout the experiment. During the entire period of auditory stimulation, participants watched a silent movie with subtitles on and were also asked to ignore the background auditory stimuli. These altogether ensure that any effect elicited by BBs would be largely implicit, i.e. not requiring overt attention. At the end of the experiment, participants rated on a 5-point scale their responses concerning the pleasantness of the background sounds, the interestingness of the movie, and their overall alertness, and finally completed a self-report questionnaire regarding personal details including musical expertise.

### Recording and pre-processing

EEG signals were recorded from sixty-four active electrodes that were placed according to the extended 10–20 system, and amplified by a Biosemi ActiveTwo amplifier. Four additional electrodes were placed around the eyes to record vertical and horizontal eye movements. The sampling frequency was 512 Hz. Biosemi system has two electrodes–active CMS (common mode sense) electrode and passive DRL (driven right leg) electrode–that together form a feedback loop representing the online reference (see http://www.biosemi.com/faq/cms&drl.htm for details on the Biosemi referencing and grounding procedures). The EEG data were later referenced offline to the average of the left and right earlobe electrodes. The data were further down-sampled offline to 256 Hz to make the file size manageable. A 0.5 Hz high-pass filter was applied to remove drifts and low-frequency artefacts. Trials containing large artefacts (< 2%) were eliminated by visual inspection from subsequent analysis. Eye-blink artefacts were corrected using Independent Component Analysis as implemented in the EEGLAB package [40]. For each auditory condition (NB and BB) of 1 min long, epochs were extracted by excluding the first and last 500 ms to eliminate transient brain responses due to the sudden onset or offset of stimulus, resulting in epochs of 59 s each.

### Data analysis

#### EEG power analysis

The spectral content of the EEG signals was estimated by the method of multitapers [41] as implemented in the Matlab function ‘pmtm’. Multitapers are sets of functions that reduce the leakage between neighbouring frequencies, thereby making it suitable for estimating the oscillatory content of neuronal signals [42]. We used a time-bandwidth parameter of 4, a 1024 point FFT (4 s window) with a frequency resolution of 0.25 Hz. We divided the broadband EEG power spectrum into five standard EEG frequency bands [37]: delta-EEG (1–4 Hz), theta-EEG (5–8 Hz), alpha-EEG (9–12 Hz), beta-EEG (13–30 Hz), and gamma-EEG (32–48 Hz); in this article, we focused our primary analysis on alpha-EEG and gamma-EEG frequency bands. The BB-related spectral power was normalized by the NB-related spectral power, and this normalization was done separately for alpha- and gamma-EEG bands, electrode, and at participant level. The normalized spectral power was expressed in dB. The auditory SSRs was estimated by averaging the EEG spectral power over alpha (gamma) frequency band when participants were stimulated by binaural beats belonging to the alpha-BB (gamma-BB). All statistical analyses were initially performed at the global level after averaging across electrodes. The statistical significance level was set at *P* < 0.05.

#### Phase synchrony analysis

For the assessment of phase synchronization (PS), it is first necessary to estimate the phases of the EEG signal, *x*_*i*_(*t*), at each electrode-*i*, a procedure that consists of two steps. First, the raw EEG data are band-pass filtered (*x*_*i*,α_(*t*)) in the frequency band of interest (say, the alpha band, α) using a finite impulsive response (FIR), zero-phase distortion filter. Then, this real valued filtered data *x*_*i*,α_(*t*) is converted into a complex-valued one,

$${\tilde{x}}_{i,\alpha }(t)=x_{i,\alpha }^{}(t)+jx_{i,\alpha }^{H}(t)$$ (1)

where *j* = $\sqrt{-1}$ is the imaginary unit and $x_{i,\alpha }^{H}(t)$ is the Hilbert Transform of the filtered data:

$$x_{i,\alpha }^{H}(t)=\frac{1}{\pi }\text{p}.\text{v}.\int_{o}^{N}\frac{x_{i,\alpha }^{}(\tau )}{t-\tau }d\tau$$ (2)

p.v. stands for Cauchy principal value. The phase of ${\tilde{x}}_{i,\alpha }(t)$, *θ*_*i α*_ *(t)* is then defined as:

$$\theta_{i,\alpha }(t)=\arctan\frac{x_{i,\alpha }^{H}(t)}{x_{i,\alpha }^{}(t)}$$ (3)

The cyclic relative phase (i.e., restricted to the interval [0,2π)) between EEG electrodes *i* and *k* (*i*, *k* = 1,…, 64) is finally obtained as:

$$\varphi_{ik,\alpha }(t)=|\theta_{i,\alpha }(t)-\theta_{k,\alpha }(t)|\mathrm{mod}2\pi$$ (4)

Assessing the degree of PS between two electrode regions comes down to estimate whether the distribution of Eq (4), which is a circular variable, is different to what would expected for two phase-independent signals. This estimation can be performed in different ways [43, 44]. An index, widely used in M/EEG analysis, is the mean phase coherence [19], also termed as Phase Locking Value (PLV); it is a measure of how homogeneously the relative phase spreads over the unit circle:

$$PLV_{ik,\alpha }=\left|<e^{j\varphi_{ik,\alpha }(\tau )}>\right|$$ (5)

where <> stands for average value and | | for absolute value. This index ranges between 0 (no phase synchrony) and 1 (perfectly synchronized in phase).

Though Eq (5) is a powerful, widely used PS indicator, it does not discriminate between zero phase lag and non-zero constant phase lag, yet two EEG signals can be phase-synchronized in both ways. Further, volume conduction effects (i.e. a single neural source affecting two or more EEG electrodes) primarily contribute to zero phase lag synchrony. Therefore, another index, phase lag index, PLI [20], has been developed to deal with this issue, by taking into account that true interaction between neural sources, as opposed to volume conduction effects, occurs with some delay [45], which in turns gives rise to a distribution of Eq (4) asymmetric around 0 (or π). Thus, PLI is defined as:

$$PLI_{ik,\alpha }=\left|\left\langle sign(\sin(\varphi_{ik,\alpha }(\text{t})))\right\rangle \right|$$ (6)

Although, from the point of view of functional connectivity analysis, it may seem enough, then, to look at Eq (6), zero lag synchronization can also be achieved if two neural sources are indirectly connected through a third one, which acts as a dynamical relay [46]. This is actually not a volume conduction effect, but rather an indirect connection between, e.g., two cortical sources through the thalamic relay [47], which would be overlooked should we only focus on PLI. Thus, both indices are complementary, with PLI estimating only direct true connections, whereas PLV is also sensitive, if any, to zero lag, indirect connections [48].

Both indices were calculated using the recently released HERMES toolbox for Matlab [49].

### Characterization of the functional brain networks

Once the degree of PS between any two electrodes was assessed by means of PLV or PLI, we had the corresponding interdependence matrix:

$$A=\left(\begin{array}{ccc} a_{11} & \cdots & a_{1n}\\ \vdots & \ddots & \vdots \\ a_{n1} & \cdots & a_{nn} \end{array}\right)$$ (7)

where *a* stands for either *PLV* or *PLI*, 0 ≤ *a*_*ij*_ = *a*_*ji*_ ≤ 1 (*i*,*j* = 1,.,*n*, *i ≠ j*), *n* = 64, and we take *a*_*ii*_ = 0.

As it is well-known, in the context of multivariate EEG analysis this matrix can be regarded as the weighted adjacency matrix of a complex network, where the electrodes are considered as nodes and the PS indices measure the strength of the links between them [21]. From this adjacency matrix, it is possible to estimate different measures that provide information about network’s structure and function.

### Thresholding

The first step in this direction consists in determining which of the values of the PS indices should be considered significant. There are different approaches for this largely unsolved question [50, 51]. A straightforward approach consists of estimating the significance of each individual link *a*_*ij*_ at a given level of statistical significance by using, e.g., bivariate surrogate data [52, 53]. The problem with this approach, however, is that one may end up with adjacency matrixes with different proportions (densities) of significant links across groups/ conditions, which may bias the values of the network measures [54], thereby giving rise to spurious statistical differences between them. This would be also the problem if we were to take a fixed threshold value *T*, so that *a*_*ij*_ was set to zero if *a*_*ij <*_ *T*. Instead, we used here the so-called *fixed density approach* [50], in which values of *a*_*ij*_ are rank ordered for each adjacency matrix, and the (*1*-*k*)*N_LINKS_ lowest values were set to zero (0<*k*<1 is the fixed density, *N*_LINKS_ = 64*(64–1)/2 = 2048 is the total number of possible links). In line with some recent studies [55, 56], we calculated all network measures (see below) for different values of *k* to check that the robustness of the results against this parameter.

### Network measures

We described the structure of the weighted undirected functional brain networks resulting from the above procedure by calculating three commonly used measures (the *strength*, the *clustering* and the *efficiency*), which characterizes network centrality, segregation and integration, respectively [21].

The average strength (*S)* of the network is defined as:

$$S=\frac{1}{N}\sum_{i\in N}S_{i}=\frac{1}{N}\sum_{i\in N}\sum_{j\neq i\in N}a_{ij}$$ (8)

where *S*_*i*_, the strength of each individual node, is the sum of weights of links connected to it, and provides a simple centrality measure that estimates the importance of the node within the network.

The clustering coefficient (*C)* quantifies the tendency of network elements to form local clusters. The presence of network clusters indicates segregated functional dependencies in the brain. In its weighted version [57], *C* is defined as:

$$C=\frac{1}{n}\sum_{i\in N}C_{i}^{}=\frac{1}{n}\sum_{i\in N}\frac{2t_{i}^{}}{k_{i}(k_{i}-1)}$$ (9)

where *C*_*i*_ is the clustering coefficient of node *i* (*C*_*i*_ = 0 for *k*_*i*_ < 2), *k*_*i*_ is the degree of node *i* and *t*_*i*_ is the sum of triangle intensities around node *i*, defined by the geometric mean of triangles around it:

$$t_{i}^{}=\frac{1}{2}{\sum_{j,h\in N}\left(a_{ij}a_{ih}a_{jh}\right)}^{1/3}$$ (10)

Finally, we measured integration in the network by means of the local efficiency [58], which can be understood as a measure of how well each sub-graph exchanges information when the index node is eliminated [59]. It is inversely related to the shortest average path lengths between nodes in the network that contains only neighbours of node *i*.

All the network measures were calculated using the Brain Connectivity Toolbox for Matlab [21].

### Statistical tests for the network measures

The comparison of brain networks can be carried out at two different levels: global and local [60]. Indeed, the three network measures described above are all calculated locally (at each node) and then averaged to get a global estimation of the corresponding feature. Correspondingly, we perform the statistical tests at these two levels.

At the global level, we used a mixed 2x2x5 factorial ANOVA, with *Group* (musicians vs. non-musicians) as between-subjects factor and *Beat* (non-binaural vs binaural beat) and *Range* (delta-BB, theta-BB, alpha-BB, beta-BB and gamma-BB) as within-subjects factors. When any of the factors (or their interaction) was significant at the *P*<0.05 level, differences were further analysed using appropriate post-hoc comparisons. The test was applied to the global measures for both types of BBs, alpha-BB and gamma-BB.

Local comparisons at the sensor level were carried out to elucidate the topography of the global differences, in those cases were the corresponding omnibus test was significant. In this case, a paired (for *Beat* and *Range*) or an unpaired (*Group*) *t*-test was applied to the three local measures at each electrode, and then corrected for multiple comparison using a Type-I False Discovery Rate (FDR) at the *q<0*.*05* level [61].

## Results

### Behavioural analysis

No statistically significant differences were found between the two groups (musicians vs. non-musicians) in terms of their reported pleasantness of the background auditory stimuli (Mann-Whitney U-test: *z* = 1.25, *P* > .05, two-tailed), interestingness of the movie (*z* = 1.75, *P* > .05), and alertness over the entire duration of the experiment (*z* = .14, *P* > 0.5).

### Power analysis

First, we calculated normalized SSRs for both alpha-BB and gamma-BB averaged across all electrodes and subsequently applied a 2x2 mixed factorial ANOVA with *Group* (musicians vs non-musicians) as a between-subjects factor and *BeatFrequency* (alpha vs gamma) as a within-subjects factor. We found a significant effect of *BeatFrequency*: SSR for alpha-BB was larger than SSR for gamma-BB (*F*(1,32) = 5.03, *P* = .03). We did not find any effect of *Group* or Group*Beat interaction (*P* > .6). Next, we investigated normalized SSR individually, as any value systematically larger than zero would suggest a significant frequency following response for that frequency band specific BB stimulation. Two separate one-sample *t*-tests were conducted and only the alpha-BB revealed a significant effect in its SSR (alpha: *t*(31) = -3.15, *P* = 0.004; gamma: *t*(31) = -1.54, *P* = .13).

**Fig 1:**
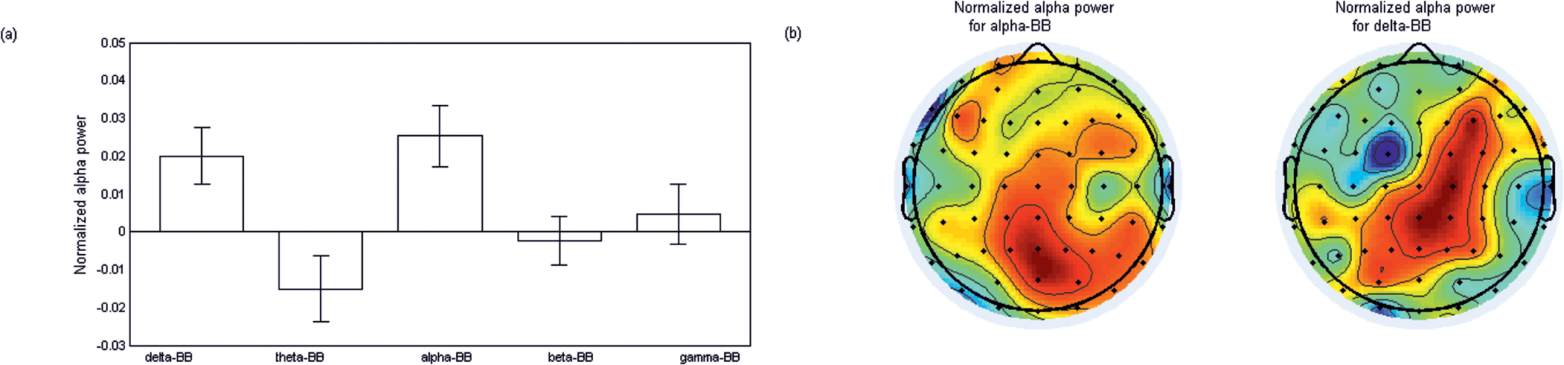
Normalized power in the EEG alpha band (9–12 Hz) during different binaural beat stimulation. BBs were grouped into five frequency bands: delta-BB, theta-BB, alpha-BB, beta-BB and gamma-BB (see *Methods* for details). Spectral power was normalized w.r.to the non-binaural beat condition presented just before the BB condition. Error bar indicates s.e.m. (b) Scalp maps of the normalized alpha power for alpha-BB and delta-BB. Results were pooled across all participants.

Next we analysed the normalized alpha-EEG power at the global level (i.e. averaged across all electrodes) separately by a 2x5 mixed ANOVA with factors, *Group* (two levels) and *BB* (five levels: delta, theta, alpha, beta and gamma). We observed a significant effect of *BB* (*F*(4,120) = 4.39, *P* = .002); the largest increase of normalized alpha-EEG power was observed for alpha-BB followed delta-BB (Fig 1(A)) and only these two showed a significant non-zero response (alpha-BB: *t*(31) = 3.15, *P* = .004, delta-BB: *t*(31) = 2.67, *P* = .01). The scalp maps of these two effects are shown in Fig 1(B).

No robust differences were observed in the normalized alpha-EEG power between the two groups except for gamma-BB for which the normalized alpha-EEG power in the musicians was higher than non-musicians (*t*(30) = 2.13, *P* = .04).

For normalized gamma-EEG power at global level, we did not observe a significant effect of *BB* (*P* > .8) but we did find a marginal effect of *Group* (*F*(1,30) = 2.4, *P* = .048) as musicians showed overall higher normalized gamma-EEG power than non-musicians.

Interestingly, when we extended our power analysis to other EEG frequency bands (delta-EEG, theta-EEG and beta-EEG, see *Methods*), we did not observe significant effect (at the level of Bonferroni corrected *P*) on the normalized SSRs (Figure A in S1 File) nor we found any significant effect of cross-frequency responses at other EEG frequency bands (Figure B in S1 File). The robust results were only found at the alpha-EEG band.

### Network analysis

**Fig 2:**
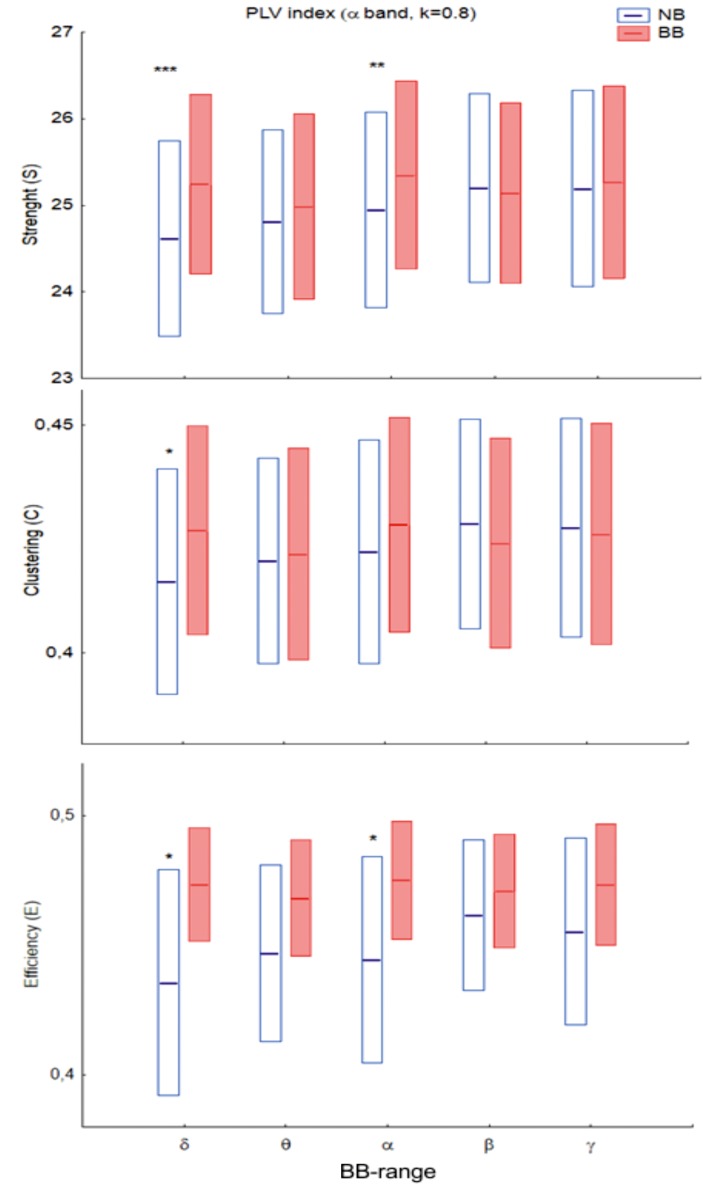
Global strength (*S*, top), clustering (*C*, middle) and efficiency (*E*, bottom) for the PLV brain network in the EEG alpha band (*k* = 0.8) for the different BB frequency ranges. Bars indicate 95% confidence interval for the mean. *Blue*: Non-binaural (NB) beat; *red*: Binaural (BB) beat. For all the indices, there was a significant interaction between the (R)ange and (B)eat factors (see *Results* for *F* and *p* values, as well as Table 1). Asterisks stand for BB-NB, differences: *: *p*<0.05; **: *p*<0.01; ***: *p*<0.001.

**Fig 3:**
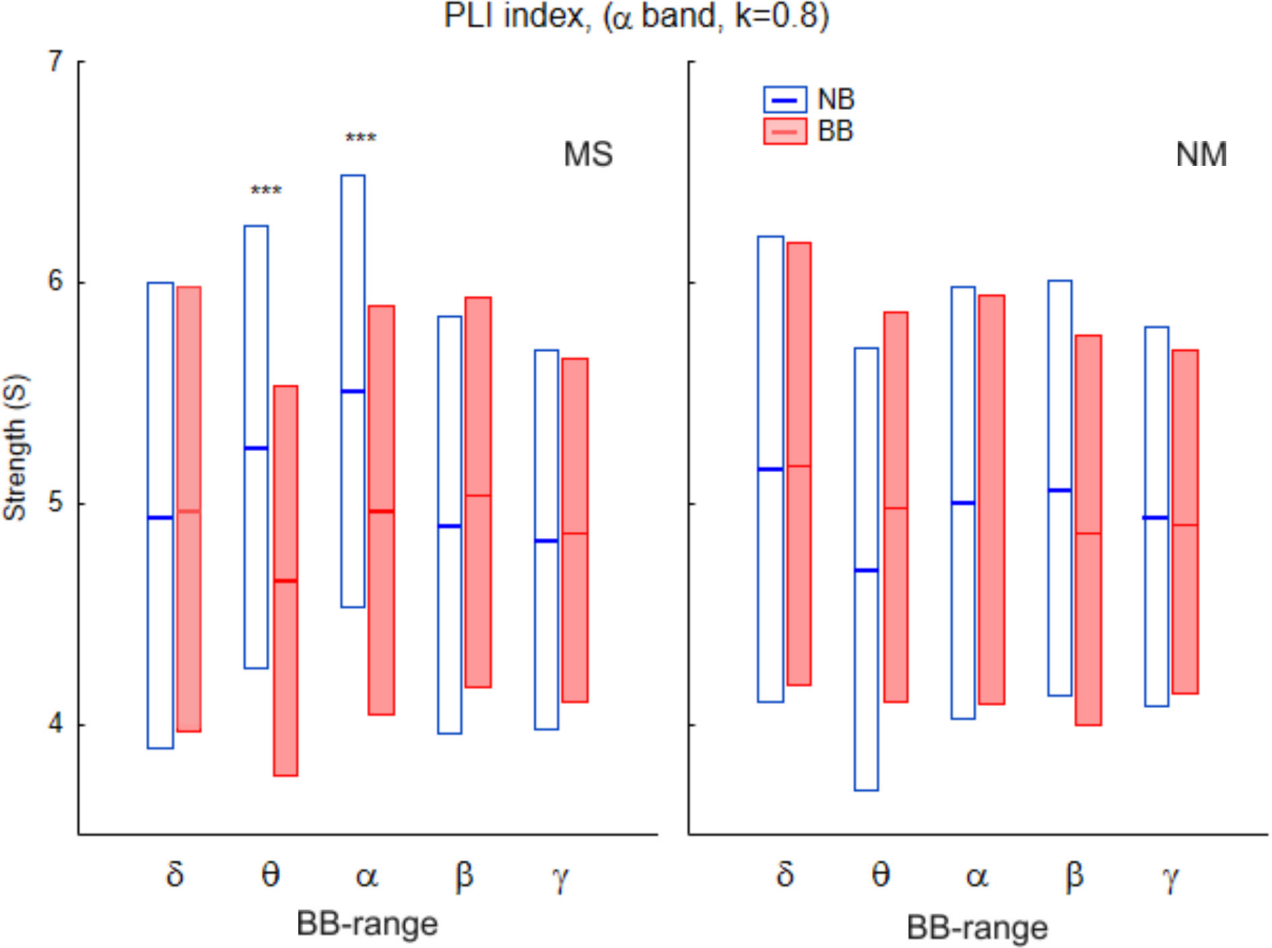
Global strength (*S*) for the PLI brain network in the alpha band (*k* = 0.8) for the different BB frequency ranges. *Left*: Musicians; *right*: Non-musicians. *Blue*: NonBinaural (NB) beat; *red*: Binaural (BB) beat. Bars indicate 95% confidence interval for the mean. There is a significant interaction between the three factors (R)ange, (B)eat and (G)roup (see *Results* for *F* and *p* values as well as Table 2). Asterisks stand for differences between BB and NB, ***: *p*<0.001.

At the global level, differences in network measures were only found for the alpha-EEG band for both phase locking value (PLV) and phase lag index (PLI), as shown in Table 1 for PLV, Table 2 for PLI, and Figs 2 and 3. In the case of the PLV, the pattern of statistical differences is complex. Thus, the main effect of *Beat* was significant for all the densities for the network strength *(S*) and the network efficient (*E*) indices, but was not present for the network clustering (*C*). In turn, the main effect of *Range* was also significant across densities for *S*, but depended on *k* for both *C* and *E*. The interaction between factors was, however, robust across all the densities analysed for both indices, although the profile was different for the PLV and the PLI. For the PLV (Fig 2), there was a significant *Beat-Range* interaction (cfr. *S*: *F*(4,120) = 5.73, *P* < 0.001; *C*: *F*(4,120) = 5.98, *P* < 0.001; *E*: *F*(4,120) = 4.53; *P* < 0.01 for *k* = 0.8), so that BB vs NB differences were only significant in the delta-BB range, with BB > NB for the three network measures. For the PLI (Fig 3), there were no main effects for any index. There was, however, an interaction among the three factors (*Beat-Range-Group*), with BB < NB for musicians in the theta- and alpha-BB ranges (cfr, *S*: *F*(4,120) = 3.9, *P* < 0.01 for *k* = 0.8).

**Table 1:** Network measures for alpha-EEG band using PLV index.

	Strength (*S*)			Clustering (*C*)			Efficiency (*E*)		
*K*	beat	F	B-R	B	R	B-R	B	R	B-R
0.8	**	*	*** (δ,α)			*** (δ)	*	*	** (δ,α)
0.6	*	**	*** (δ)		*	*** (δ)	*		*** (δ)
0.4	*	***	*** (δ)		**	* (δ)	*		** (δ)

Strength (*S*), clustering (*C*) and global efficiency (*E*) for the PLV index as a function of the density of network links, *k*. The results are for alpha-EEG band. Asterisks stand for differences for the main effects (B)eat (*Nonbinaural* vs. *Binaural*) and (R)*ange* (low frequency delta-, *δ—*, to high frequency, *γ-*BB), as well as the interaction between them (B-R)

*: *p* < 0.05

**: *p* < 0.01

***: *p* < 0.001. In the B-R column, Greek letters indicate the BB range for which BB—NB differences were significant.

**Table 2:** Network measures for alpha-EEG band using PLI index.

Density, *k*	Strength, *S*	Clustering, *C*	Efficiency, *E*
0.8	B-R-G: **	B-R-G: **	B-R-G: **
0.6	(θ, α)	(θ, α)	(θ, α)
0.4	BB<NB in MS	BB<NB in MS	BB<NB in MS

Strength (*S*) Clustering (*C*) and global efficiency (*E*) for the PLI index for different densities of network links. The results are for alpha-EEG band. Asterisks stand for differences for the interaction between the three factors (B)eat (Nonbinaural vs. Binaural), (R)ange (*δ*-BB to γ-BB band) and (G)roup (*Musicians*, *MS vs*. *Nonmusicians)*, *B-R-G;*

**: *p*<0.01. Greek letters indicate the BB range for which BB—NB differences were significant. Note that the differences and the bands were robust across all the density links considered.

**Fig 4:**
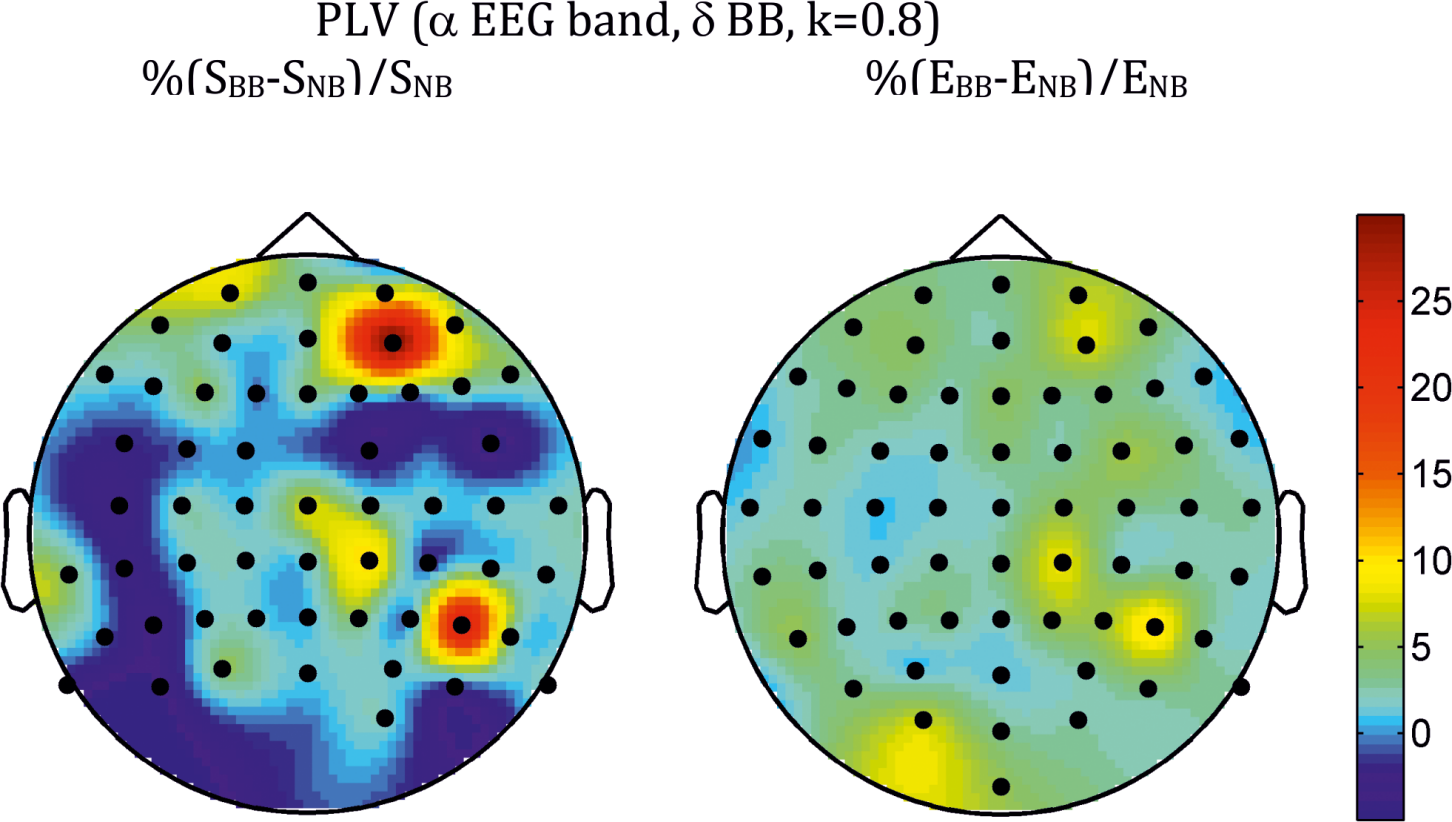
Average across all subjects of the topographic distribution of differences in strength (*S*, *left*) and efficiency (*E*, *right*) between BB and NB for the PLV index (alpha EEG band, *k* = 0.8, delta-BB). Bullets indicate the position of electrodes for which differences were significant at the *q* < 0.1 level (FDR type I) using a paired *t*-test.

**Fig 5:**
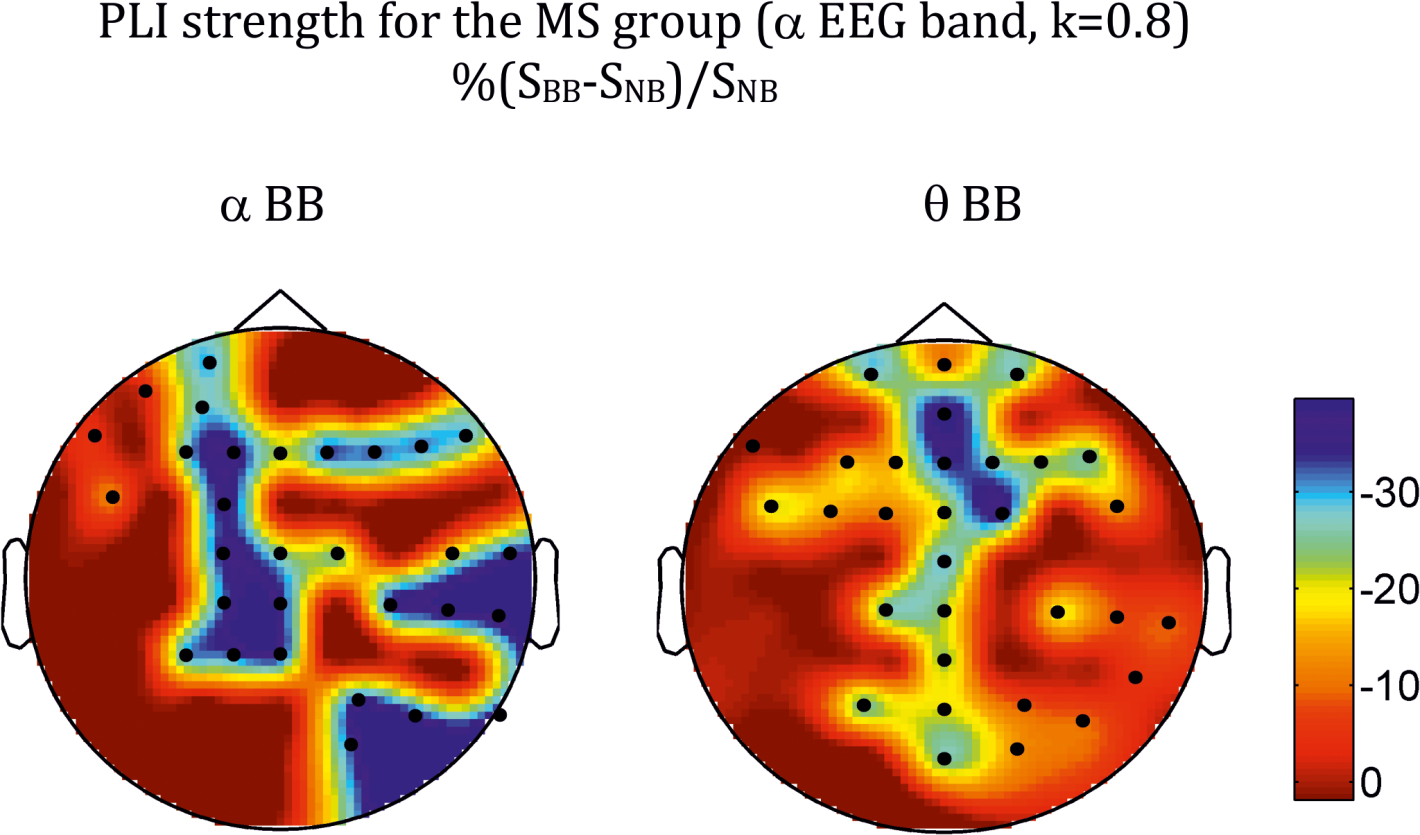
Average across all subjects in the musicians group of the topographic distribution of differences in strength (*S*) between BB and NB for the PLI index (alpha EEG band, *k* = 0.8). *Left*: theta-BB; *Right*: alpha-BB. Bullets indicate the position of electrodes for which differences were significant at the *q* < 0.1 level (FDR type I) using a paired *t*-test.

**Fig 6:**
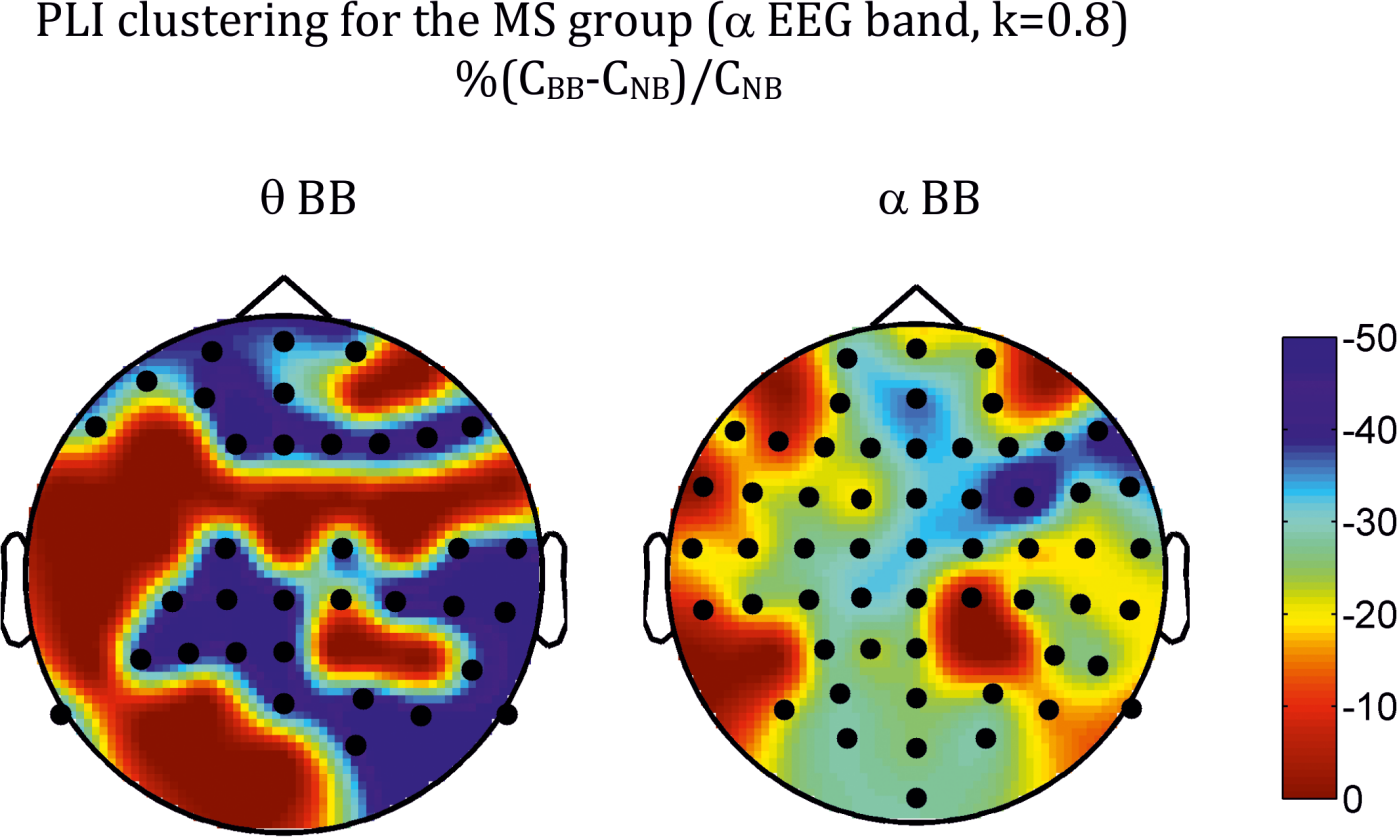
As in Fig 5 but for the clustering index (*C*).

The topologies of the differences at the electrode level are shown in Fig 4 for PLV and Fig 5 for PLI. In both cases, they correspond to the density *k* = 0.8, but are robust across the other values of *k* analysed. For the PLV, the topology is strikingly similar for the three measures, with differences spreading over many electrodes, the greatest ones showing clear right hemisphere lateralization and antero-frontal and centro-parietal localization, with both *C* (not shown) and *E* showing also a left parieto-occipital patch. For the PLI, however, the pattern is different for *S* (Fig 5) than for the other two measures (Fig 6 for *C*). Thus, differences in *S* were mainly concentrated along the midline electrodes (both theta- and alpha-BB range), with an additional right temporo-occipital patch in the alpha band; for *C* and *E*, although differences showed a certain trend for right hemisphere lateralization (more posterior for the theta than for the alpha-BB range), they were widespread over many electrodes.

## Discussion

The current study investigated the relationships between BB stimulation and large-scale neuronal oscillations and synchrony, and the potential influence of musical training on these relationships. We investigated the alpha and gamma band oscillations in the EEG signals against a range of BB stimulations; both local (in terms of spectral power), long-distance (in terms of phase synchronization) neuronal synchronization were investigated in addition to characterizing the underlying brain network in these two bands of neuronal oscillations. Finally, we also investigated the impact of musical expertise on the brain responses to BB stimulations.

### Power analysis

We have identified two types of oscillatory responses: (i) narrow band or frequency following responses suggesting a kind of neuronal entrainment or a linear relationship to the beat frequency, and (ii) broadband or cross frequency responses suggesting a kind of nonlinear relationship.

As predicted, we found significant entrainment in the EEG-alpha band across participants; the alpha band EEG power was significantly enhanced during alpha-BB stimulation. Early research literature indicates that presentation of a visual rhythmic stimulus (i.e. a flickering light) at alpha band frequencies lead to a robust enhancement of EEG power in alpha and harmonic or sub-harmonic frequencies of alpha [62, 63], known as photic driving. However, the evidence for the similar driving response caused by auditory rhythmic stimuli has been mixed [15, 64–67]. The reported absence of BB-related entrainment could be due to several methodological issues, such as using a higher *f*_*b*_ of 400–500 Hz instead of 100–200 Hz, single BB stimulation instead of a range of stimulation or sparse spatial sampling of brain regions. By systematically varying the BB frequency and using high density EEG recording followed by carefully constructed statistical analysis, we here demonstrated the role of alpha band in auditory driving as well.

Alpha, being the dominant neuronal oscillations in the human brain, is classically considered as the 'idling rhythm' [68, 69]. However, a large body of recent evidence suggest that alpha band oscillations play an active and important role in both task-specific and ongoing information processing (see [70] for a review). Our alpha effect was largest over posterior parieto-occipital electrode regions, roughly overlying visual cortices. Previous research suggests that parieto-occipital alpha power increases with anticipatory attentional allocation mechanisms [71] and distractor suppression [72]. Altogether, our results are also aligned with behavioural studies suggesting that alpha band rhythmic stimulation could enhance attentional [73] and memory [74, 75] performance, and reduce the sensation of unpleasant stimuli [76].

In terms of cross frequency responses, alpha power was significantly enhanced by delta-BB stimulations, with the effect being predominant over parietal electrode regions extending to right frontal regions. By using a variety of auditory stimuli with and without rhythmic component, it was shown that listening to rhythmic stimuli, as compared to non-rhythmic ones, was associated with higher EEG alpha power, and further, the auditory envelope was synchronized with the alpha oscillations [65]; this suggests some kind of frequency selectivity of brain oscillations against auditory or visual stimulation [77], something that has been put forward as one of the possible models of an alpha generator [78]. The spatial locations of this effect, the fronto-parietal regions, are aligned with a previous study in which auditory SSRs were evoked by BBs of 4 Hz and 6.66 Hz [7]. A more recent fMRI study has found that acoustic exposure to a more isometric rhythmic sequence was associated with activations in the parieto-frontal brain regions, including dorsal premotor cortex and supplementary motor cortex [79]. An isometric sensation can also be perceived while listening to BBs due to the interaural phase differences (IPD). Bengtsson *et al*. [79] suggested that activation in these brain areas might reflect the temporal sensory prediction.

Further, the BBs in lower frequencies (e.g. in delta, theta and alpha frequency bands) often elicit a subjective sensation of a moving ‘image’ that swaps from one side to another side (laterally) within the head [80]. This internal sensation of a moving object from place to place in the head may partly explain the engagement of the fronto-parietal brain network. In fact, an ERP study showed that the sensation of a moving sound as caused by the interaural time differences (ITD) was associated with right inferior parietal and bilateral inferior frontal activities [81]. Nevertheless, further examination is needed to establish the link between consciously perceived sensation of motion induced by BB stimulation and such brain activity patterns.

Similar evidences of cross-frequency function of BBs were also found by Kasprzak [36], who examined the effect of BB in 10 Hz. Results revealed higher activation of the EEG 10 Hz strength signal. Interestingly, averaging amplitudes of spectral density into frequency bands lead to a significant decrease for alpha and beta bands and increase for theta frequency bands. He suggested that this phenomenon of alpha suppression in parallel with a 10 Hz follow-up effect might be a natural reflex of the nervous system to be tuned to a strong external stimulus. Cross-frequency results were also reported by Atwater [82] who found a reduction of occipital alpha when stimulated by a complex pattern of BBs mainly in delta band. These results may also give an explanation to our findings underling the non-linear dynamic function of the brain under various BB stimulations. Moreover, encoding of BBs may also be a process of a more complicated mechanism involving both a cognitive process, as Karino *et al*. [7] suggested, and an encoding of the IPD in the ascending auditory pathway [3, 4, 10].

Comparisons between musicians and non-musicians revealed higher alpha power over the frontal and parietal regions for gamma-BB stimulations (figure not shown). Previous fMRI studies revealed that frontal and parietal lobes show higher activation bilaterally in musicians as compared to non-musicians during passive listening to piano melodies [83]. Similarly, Meister *et al*. [84] reported bilateral activations of the parietal-frontal network during music performance and musical imagery. An additional explanation why alpha power increases in musicians across gamma-BB stimulation may be that stimuli higher than 30 Hz (gamma-BB stimulation) falls into the audible range (20 Hz—20 kHz) of the humans’ auditory system and is effortlessly recognizable as a bitonal instead of binaural sensation. The frequency intervals, which resulted from the mismatched frequency between both stimuli, are easier to be detected and distinguished as two different tones, especially for well-trained musicians [85]. The Δ*f* which results (left ear: 200 Hz and right ear: 200+*f*_*bb*_ Hz [*f*_*bb*_ = 30–48 Hz]) creates a sensation of a ± minor 3^rd^ musical interval of the well-tempered tuning system (minor 3^rd^; ratio: *f*6/5 _(*f-*reference = 200 Hz)_ = ~240 Hz, scale pitch names: near to G_3_ and B^♭^_3_). Therefore this bitonal sensation could elicit higher frequency selectivity for musicians who are exposed much longer to musical sounds, and therefore could lead to higher alpha power across gamma-BB stimulation. Musicians also showed marginally higher overall gamma power across BB stimulations. This is aligned with previous findings that musicians present larger gamma band oscillations [86] and synchronizations [29] while listening to musical stimuli.

### Network analysis

To our knowledge, this is the first study on characterizing the cortical network pattern when stimulated by binaural beats. Our second prediction of finding a greater network in the gamma band oscillations for musicians as compared to non-musicians was not supported. Instead we have found that the differences between the groups and also the effects of binaural beat were found in the alpha band EEG only.

Before we interpret our findings of network analysis, we would like to include some remarks on analysis methodology. It is not exaggerated to state that the network analysis based on graph theoretic methods has been at the forefront of the current neuroimaging research and it has become customary in multivariate EEG studies [87–89]. Despite the extensive literature in the subject, however, there are still many important open questions in the field [50]. Among them, we can mention the choice between weighted and unweighted networks, choosing a threshold to consider an edge significant, the effect of network size, which network index best characterizes between group (or tasks) differences, how to deal with the multiple comparison problem and even which functional connectivity index is the right one for a given problem [51, 54, 90]. Here, we had three popular network indices characterizing different network properties (strength, clustering, efficiency [21]). Moreover, in line with recent EEG studies [55, 56], we have used weighted networks, which provide additional information on edge weights as compared to Boolean networks, and have checked that the statistical differences are consistent for a wide range of degree densities (see Tables 1 and 2), both at the global and at the local level, at which we corrected for multiple comparisons by means of the type I FDR algorithm. One important feature of our study, however, is that we analysed the functional networks obtained from *two* different PS indices, whereas the usual approach entails the use of either the PLV *or* the PLI. At first sight, it may seem a little odd to use them jointly, since PLI was developed as a kind of refined version of the PLV, robust against to volume conduction due to its insensitiveness to zero lag correlations [20, 44]. Yet we think (and the results seem to agree with us) that there are good reasons to include both the PLI and the PLV in the analysis (see also [49] for a recent example), because, as commented in the *Methods* section, both indices provide complementary rather than redundant information. Indeed, whereas PLI is in principle the right choice for M/EEG graph connectivity analysis as it focuses on lagged (direct) correlations [91, 92], it may miss true, zero lag cortico-cortical connectivity through a common relay (i.e., the thalamus [93–95]), which could be picked up by the PLV. Thus, by analysing both functional brain networks (the “true” one derived from PLI and the one derived from PLV, which may be affected by volume conduction effects, but includes all type of connections), we can be sure to gather most of the information available from our data in the form of functional connectivity patterns. Indeed, recent results comparing the application of PLV to PS indices robust to volume conduction, clearly suggest [96, 97] that PLV may provide additional information not present in other indices, whereas the use of advanced pre-processing methods such as the Laplacian to work with current source densities instead of voltages does not solve the volume conduction problem either [98].

In concrete terms, our results showed that both indices agreed in showing that statistical differences are only present in the alpha frequency band and for the lower range of binaural beats (delta to alpha), with a slight right hemisphere dominance. But the network of direct cortico-cortical connections (as assessed by the PLI) presented differences only for the group of musicians (Table 2, Figs 3, 5 and 6), with a decrease in overall connectivity, clustering and efficiency during the processing of the binaural beat. Yet this decrease ran in parallel to a (group-unspecific) increase of all network indices for the PLV network (Table 1, Figs 2 and 4), although in this case an alternative explanation might be that during BB there might be an enhanced volume conduction effects due to increased deep source activation. Taken together, these results indicate that processing of low range (delta to alpha) binaural beats significantly increased zero lag (i.e., mediated by deeper relay neural sources) phase synchrony between cortical areas (and possibly also the overall oscillatory activity of these deeper sources) in the alpha band for all subjects regardless of the level of training level in sound processing, but a concomitant reduction in direct cortico-cortical alpha band connectivity is indeed related to the level of expertise in sound processing.

### Limitations and future extension of the research

We would like to mention here a few practical remarks. First, sound intensities of this study were not kept constant across participants, instead were self-adjusted by participants at individual level of comfort. Higher intensities may generate possible cross-hearing (bone or fluid conduction) which may affect the presented data [38]. Second, the mismatch frequency was always presented to the right ear, thereby limiting any interpretation on lateralization effect [99]. However, this stands for all of our participants, so any group related effects are unlikely to be affected by this presentation bias. Third, our musicians group was heterogeneous in terms of type of instruments played and years of musical experience, which could explain the marginality of the reported group related effects. Future studies could be focused on different subcategories of musicians such as those with absolute vs. relative pitch [100, 101], years of experience [102], type of instrument played [16, 103], or even gender [104, 105]. Fourth, in addition to musical expertise, the two groups may also differ across other dimensions, like personality. Previous research does suggest that personality traits may partly explain individual differences in the entrainment induced by BB stimulation [106, 107], though a recent study did not find any significant relationship between neuronal entrainment and personality variables against a short (2 min) BB stimulation [15]. Finally, our results revealed the neural correlates of BB processing, but due to the lack of behavioural measures related to the reported BB illusion, the specificity and potential implications of the reported effects are less than explicit. On the other hand, these effects could be considered as markers of implicit processing of BB stimuli.

## Conclusions

In summary, the present study revealed that binaural beat stimulation can modulate the strength of neuronal oscillations and synchrony obtained noninvasively from the scalp EEG. Various regions of the brain, such as frontal, temporal and parietal lobes, seem to be involved in the procedure of binaural auditory mismatched frequencies stimulations. Alpha brain oscillations appear to be the most prominently entrained to the perceived binaural beat illusion, and further, alpha band network appears to be significantly modulated by low frequency binaural beats.

## Supporting Information

S1 File BB-related SSRs and cross-frequency responses. **Figure A**: Normalized SSRs in each frequency band across all participants, error bars indicate 99% confidence intervals. A significant effect was observed during alpha-BB stimulation. **Figure B**: Cross-frequency responses against BB stimulations, error bars indicate 99% confidence intervals. A significant enhancement of alpha-EEG power during delta-BB stimulation was found.
(DOCX)
