# Supplementary material for: Electrical Brain Responses to an Auditory Illusion and the Impact of Musical Expertise
Source: PLoS One. 2015 Jun 12;10(6):e0129486. doi: 10.1371/journal.pone.0129486 (PMC4466486; doi:10.1371/journal.pone.0129486)
Supplement: S1 File — Figure A: Normalized SSRs in each frequency band across all participants, error bars indicate 99% confidence intervals. A significant effect was observed during alpha-BB stimulation. Figure B: Cross-frequency responses against BB stimulations, error bars indicate 99% confidence intervals. A significant enhancement of alpha-EEG power during delta-BB stimulation was found. (DOCX) [file pone.0129486.s001.docx]

**Electrical Brain Responses to an Auditory Illusion**

**and the Impact of Musical Expertise**

**Christos I. Ioannou^1,2^, Ernesto Pereda^3,4^, Job P. Lindsen^1^, Joydeep Bhattacharya^1,*^**

^1^Department of Psychology, Goldsmiths, University of London, London, United Kingdom

^2^Institute of Music Physiology and Musicians' Medicine, Hannover University of Music, Drama and Media, Hanover, Germany

^3^Electrical Engineering and Bioengineering Group, Department of Industrial Engineering, University of La Laguna, Tenerife, Spain

^4^Institute of Biomedical Technology (CIBICAN), University of La Laguna, Tenerife, Spain

*j.bhattacharya@gold.ac.uk

**Additional Power Analysis**

We calculated normalized SSRs for all five EEG frequency bands (Figure A). Any value systematically larger than zero would suggest a significant frequency following response for that frequency band specific BB stimulation. Five separate one-sample *t*-tests were conducted (Bonferroni-corrected *P*, *P_corr_* = 0.01) and only the alpha-BB revealed a significant effect in its SSR. Although some of the effect sizes of the SSRs in other frequency bands were considerable (i.e. delta-BB and gamma-BB), these effects turned out to be non-significant (*P* > *P_corr_*) due to large variability across participants.


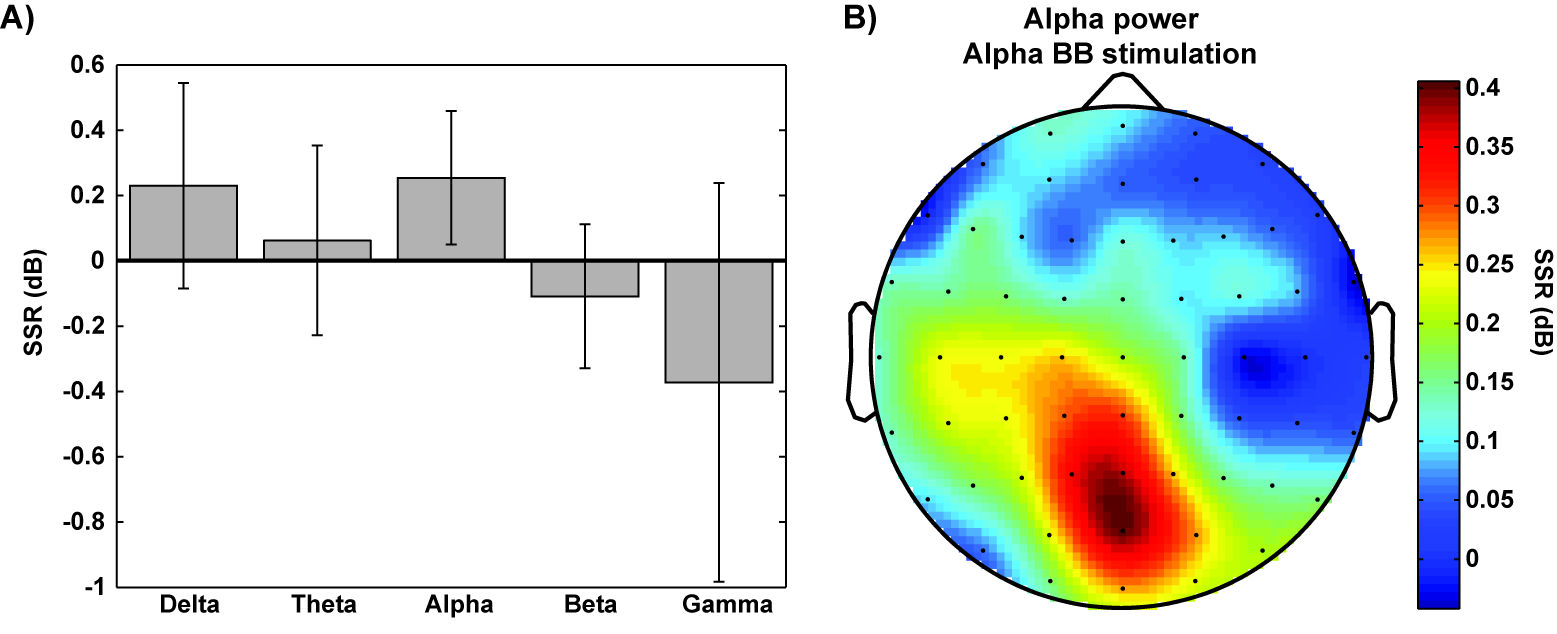


**Figure A**. Normalized SSRs in each frequency band across all participants, error bars indicate 99% confidence intervals. A significant effect was observed during alpha-BB stimulation.

Next we investigated the cross-frequency responses against BB stimulation and the results are shown in Figure B. For each EEG frequency band specific BB stimulation, we conducted four separate one-sample *t*-tests (*P_corr_* = 0.0125). The only significant effect was found in the alpha-EEG power for low frequency delta-BB stimulation (*t*_31_ = 2.67, *P* = 0.012 < *P_corr_*, *d* = .47); for BB stimulation at the rest of the frequencies, we did not find any cross frequency effect surviving the Bonferroni-corrected level of significance.


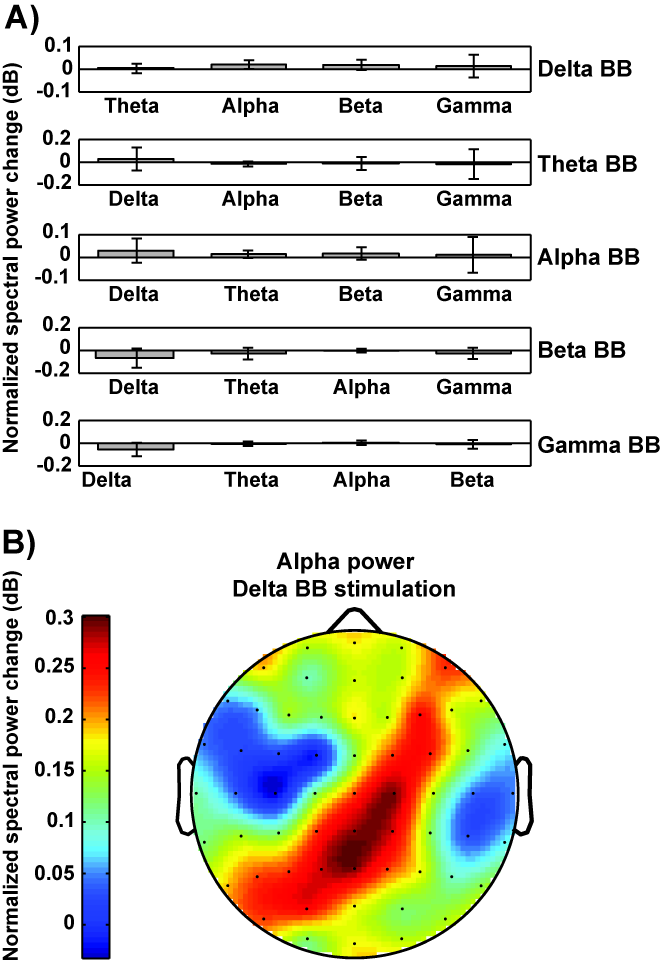


**Figure B.** Cross frequency responses against BB stimulations, error bars indicate 99% confidence intervals. A significant enhancement of alpha-EEG power during delta-BB stimulation was found.
